# External Quality Assessment Evaluating the Ability of Dutch Clinical Microbiological Laboratories to Identify *Candida auris*

**DOI:** 10.3390/jof5040094

**Published:** 2019-10-07

**Authors:** Jochem B. Buil, Henrich A. L. van der Lee, Ilse Curfs-Breuker, Paul E. Verweij, Jacques F. Meis

**Affiliations:** 1 Department of Medical Microbiology, Radboud University Medical Center, 6525GA Nijmegen, The Netherlands; Henrich.vanderLee@radboudumc.nl (H.A.L.v.d.L.); Ilse.Curfs-Breuker@radboudumc.nl (I.C.-B.); paul.verweij@radboudumc.nl (P.E.V.); jacques.meis@gmail.com (J.F.M.); 2 Center of Expertise in Mycology Radboudumc/CWZ, 6525GA Nijmegen, The Netherlands; 3 Department of Medical Microbiology and Infectious Diseases, Canisius-Wilhelmina Hospital (CWZ), 6532SZ Nijmegen, The Netherlands

**Keywords:** *C. auris*, MALDI-TOF MS, misidentification

## Abstract

Background: *Candida auris* is a yeast that is causing nosocomial outbreaks in healthcare facilities around the world. There is a risk of the misidentification of *C. auris* with matrix-assisted laser desorption ionization-time of flight mass spectrometry (MALDI-TOF MS)—when libraries are used that lack *C. auris* spectra, or when conventional biochemical methods are used. Methods: We conducted an external quality assessment to evaluate the ability of Dutch clinical microbiological laboratories to identify *C. auris*, and to raise awareness about the risk of misidentification. Results: 35/47 participating laboratories were able to identify *C. auris* correctly. Only 2/14 labs that potentially misidentified *C. auris* with their primary identification methods specified that they would perform additional tests to exclude *C. auris* when appropriate. 45/47 labs used MALDI-TOF MS systems to identify *Candida* species. Conclusions: There was a lack of awareness about the potential misidentification of *C. auris* in many labs that used MALDI-TOF MS with libraries that lacked *C. auris* spectra, and labs that used Vitek 2. However, as the currently available MALDI-TOF MS libraries in The Netherlands contain several *C. auris* spectra, we expect that currently almost all participating laboratories are able to identify *C. auris* correctly, as 45/47 participating laboratories use MALDI-TOF MS as their primary yeast identification method.

## 1. Introduction

*Candida auris* is an ascomycete yeast that is causing nosocomial outbreaks in healthcare settings around the world [1,2,3,4,5,6,7,8,9,10,11,12,13,14,15,16,17]. The yeast is an emerging pathogen and is considered a serious global health threat, due to its common multidrug-resistant phenotype and ability to cause outbreaks in health care settings [18,19,20,21]. The lack of its early correct identification, and the failure to implement the immediate contact precautions of infected or colonized patients, are the key conditions in need of consideration to prevent hospital transmissions and silent outbreaks. Reliable identification of *C. auris* is possible by sequence analysis of the D1/D2 region of the large subunit and the internal transcribed region of the ribosomal RNA, or by matrix-assisted laser desorption ionization-time of flight mass spectrometry (MALDI-TOF MS) [22,23,24,25,26]. However, there is a risk of misidentification when biochemical assays are used, or when the reference libraries lack *C. auris* spectra in their databases when MALDI-TOF MS systems are used. We organized an external quality assessment (EQA), to evaluate the ability of Dutch clinical microbiology laboratories to correctly identify *C. auris* isolates, and to raise awareness about this pathogen’s potential to cause hospital outbreaks, and also the possibility of misidentifications. 

## 2. Materials and Methods 

The EQA panel consisted of six samples: Three samples containing *C. auris* isolates (V238-35, V238-38, and V238-40), two with *C. haemulonii species* complex (V238-33, containing both *C. haemulonii/pseudohaemulonii* and V238-39 *C. duobushaemulonii*), and V238-37 *Candida lusitaniae*. The isolates were identified by sequence analysis of the internal transcribed spacer region 1 and 2 [24]. Isolates were grown on Sabouraud dextrose agar plates and transferred to Sabouraud dextrose agar slants in a screw-cab tube, and incubated for 24 h at 35 °C. Then, in February 2018, the agar slants were sent to 63 clinical microbiology laboratories in The Netherlands. A questionnaire was added, containing a series of questions about the available methods for the identification of yeasts, and on which isolates these methods would be used. Laboratories were asked to report their results by returning the questionnaire and identification results by mail. The Center of Expertise in Mycology Radboudumc/CWZ analyzed the results, and returned the conclusions anonymously to all participants. 

## 3. Results

### Identification 

**Figure 1:**
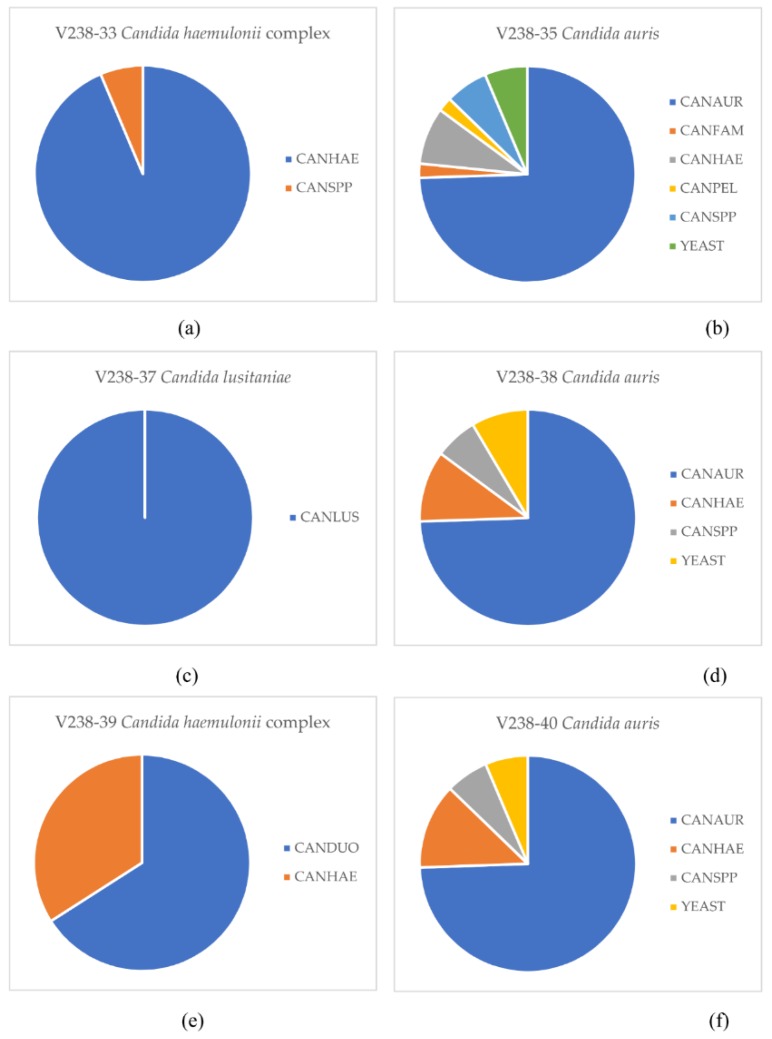
Identifications provided by 46 Dutch clinical microbiology laboratories for a panel of six *Candida* isolates. (**a**) *C. haemulonii* complex (containing both *C. haemulonii* and *C. pseudohaemulonii*); (**b**) *C. auris*; (**c**) *C. lusitaniae*; (**d**) *C. auris*; (**e**) *C. haemulonii* complex (*C. duobushaemulonii*); and (**f**) *C. auris*. CANAUR: *C. auris*, CANDUO: *C. duobushaemulonii*, CANFAM: *C. famata*, CANHAE: *C. haemulonii*, CANPEL: *C. pelliculosa*, CANLUS: *C. lusitaniae*, CANSPP: *Candida* species.

Seven university hospital laboratories and 40 teaching and non-teaching hospital laboratories took part in the EQA. Thirty-five of 47 (74%) laboratories correctly identified the three *C. auris* isolates. The *C. auris* isolate V238-35 was wrongly reported as *C. haemulonii* by 4 (9%) laboratories, as *C. famata* and *C. pelliculosa* by one (2%) laboratory, and as *Candida* species or yeast by 6 (13%) laboratories. *C. auris* isolate V238-38 was misidentified as *C. haemulonii* by 5 (11%) laboratories, and as *Candida* species or yeast by 7 (15%) laboratories. *C. auris* isolate V238-40 was misidentified as *C. haemulonii* in 6 (13%) laboratories, and as *Candida* species or yeast by 6 (13%) labs. The mix of *C. haemulonii* and *C. pseudohaemulonii* C238-33 was identified as either *C. haemulonii* or *C. pseudohaemulonii* by 44 (94%) laboratories, and as *Candida* species by 3 (6%) laboratories. *C. duobushaemulonii* isolate V238-39 was correctly identified by 31 (66%) laboratories, and wrongly as *C. haemulonii* by 16 (34%). All laboratories correctly identified *C. lusitaniae* isolate V238-37 (Figure 1). Thirty-two of 33 (97%) laboratories that used the Bruker MALDI Biotyper MALDI-TOF MS system (Bruker daltonics, Bremen, Germany) correctly identified the three *C. auris* isolates; while this was the case for three of 12 (25%) laboratories that used the bioMérieux Vitek MS MALDI-TOF MS system (bioMérieux. Vitek MS, Marcy-l’Étoile, France) (Table 1). However, three laboratories that used the Vitek MS system indicated that additional methods were used to identify *C. auris*; two labs performed ITS sequencing and one lab used qPCR. Another lab indicated that they would send unidentified *Candida* isolates to a reference laboratory for identification.

Six out of seven (86%) clinical microbiology laboratories of university medical centers correctly identified the three *C. auris* strains, while 30 of 40 (75%) teaching and non-teaching hospitals correctly identified the isolates (Table 2). 

The two laboratories that did not use MALDI-TOF MS for the identification of yeast were not able to identify the *C. auris* isolates with their available methods. Both laboratories indicated that the unidentified isolates would have been sent to another laboratory for identification. However, both used Vitek 2 for identification, and this method identified the *C. auris* isolates as *C. haemulonii*. 

## 4. Discussion

The results of the EQA showed that 12 of 47 (25%) clinical microbiology laboratories in The Netherlands were not able to identify *C. auris* isolates correctly, at the time that this EQA was conducted. The ability to correctly identify *C. auris* depended on the manufacturer of the MALDI-TOF, with those laboratories using the Bruker MALDI Biotyper being the most accurate. Only three laboratories that used the bioMérieux Vitek MS system reported a correct identification. However, these laboratories did not use the bioMérieux Vitek MS for identification of *C. auris* to the species level, but used additional identification methods like qPCR, or sequence analysis. The difference between the results of the bioMérieux Vitek MS and the Bruker MALDI Biotyper systems can be explained by the reference databases that were available at the time the EQA was conducted. The CE-IVD approved library version 4.0 of the Bruker MALDI Biotyper IVD system, available in Europe at that time, contained three *C. auris* spectra, while the bioMérieux Vitek MS libraries did not. However, the Knowledge Base V. 3.2 update contains several *C. auris* spectra, and was CE-IVD marked in June 2018, and is now widely used [27,28].

When the EQA was conducted, several publications reported that the bioMérieux Vitek MS misidentified *C. auris* isolates as *C. lusitaniae, C. haemulonii*, or *C. albicans*, or resulted in no identification, when libraries without *C. auris* spectra were used [29,30]. However, when using the RUO database (SARAMIS spectra base) that included *C. auris* spectra, it should be possible to identify *C. auris* correctly [26]. Of the labs that used bioMérieux Vitek MS, three labs were able to correctly identify *C. auris* with the methods available in their labs, but only two labs reported that they would perform additional testing when potential misidentifications with Vitek MS were possible (e.g., identification as *C. haemulonii*, or *C. lusitaniae*, or no identification). Two other labs specified that they would send isolates from blood cultures or sterile materials to a mycology reference laboratory for identification when bioMérieux Vitek MS was unable to provide a species identification. In the latter scenario, *C. auris* would be potentially missed when cultured from non-sterile samples. However proper identification is relevant for infection control, as *C. auris* colonization is an important risk factor for transmission [31,32]. Furthermore, *C. auris* isolates misidentified as *C. haemulonii* or *C. lusitaniae* would have been reported as identified. These results indicate that several laboratories in The Netherlands were not aware of the potential risk of misidentification of *C. auris* with bioMérieux Vitek MS, at the time the EQA was conducted. 

An important objective of this EQA was to raise awareness about the potential misidentification of *C. auris* and its ability to cause nosocomial outbreaks, as early recognition is pivotal to limit the unrecognized spread of *C. auris* in the hospital environment [3,9,31,32]. As similar to the results of a laboratory survey in Belgium that was conducted during the same period, the results of this EQA indicated that not all laboratories were aware of the potential risks of *C. auris*, nor of the limitations of their yeast identification protocols [28]. We believe that our EQA contributed to increasing awareness, and that together with the updated databases of the MALDI-TOF, unexpected cases of *C. auris* would be identified. 

In addition to the possible misidentification with MALDI-TOF MS, several reports indicate that the use of conventional biochemical methods, and the use of bioMérieux Vitek 2 YST ID (Vitek 2) cards, may potentially result in misidentification. The API 20C AUX clinical yeast system has been reported to misidentify *C. auris* as *C. sake*, or *Rhodotorula glutinis*, when the isolate has a negative urease reaction [24,33,34]. One participating laboratory reported that they used the API 20C AUX system for yeast identification, but only when the bioMérieux Vitek MS would not be able to identify the yeast species. The Vitek 2 may potentially identify *C. auris* isolates as *C. duobushaemulonii*, *C. haemulonii*, or as *C. famata* [4,22,29,33,34,35,36,37,38,39]. Several participating laboratories reported the use of the Vitek 2 system, but most would only use it when the MALDI-TOF MS failed to speciate the isolates. Only two labs used the bioMérieux Vitek 2 system as a primary yeast identification method. The updated 8.01 bioMérieux Vitek 2 YST ID system does contain *C. auris* in its database, and thus may identify *C. auris* correctly. However, a recent multicenter study showed that only 52% of 35 *C. auris* isolates from different clades were correctly identified. Interestingly, differences in the performance among clades were observed. These populations are commonly referred to as the South Asian (I), East Asian (II), African (III), and South American (IV) clades. All isolates from the South American (clade IV) (*n* = 8) were correctly identified, whereas 74% (*n* = 13) of the South Asian (clade I), and only 7% (*n* = 10) and 0% (*n* = 4) of isolates from the African (clade III) and East Asian (clade II) were correctly identified [40]. Thus, regardless of having *C. auris* in its database, this study showed that the identification of some clades of *C. auris* remains problematic, and identification of *C. auris* and *C. duobushaemulonii* should trigger additional testing to exclude *C. auris*. 

Although not used by the participants in this study, several other methods for identification of yeast are known to misidentify *C. auris* isolates. However, these methods are still used by a significant number of laboratories elsewhere in the world. The misidentifications by other methods have been extensively reviewed elsewhere [25,41]. 

Whole genome analysis suggests the emergence of five different *C. auris* clades [42,43]. It seems that by using reference databases containing more *C. auris* spectra from different clades, most isolates can be identified correctly with MALDI-TOF MS [44]. Thus, it is likely that with the currently updated Bruker MALDI Biotyper and bioMérieux Vitek MS reference libraries, that contain several *C. auris* spectra, excellent identification may be achieved. However, a multicenter study with bioMérieux Vitek 2 showed that the performance may vary among *C. auris* clades for this method [40]. Whether or not the performance of MALDI-TOF MS also varies between the *C. auris* clades has not been studied extensively. With the Bruker MALDI Biotyper and a library that contained only three *C. auris* spectra, a study showed that all 90 (Indian) isolates could be identified as *C. auris* (South Asian clade I) [22]. A second study showed that 83.6% and 98.4% of 61 isolates could be identified as *C. auris*, with cutoff scores of >1.7 and <1.7, respectively (East Asia clade II) [45]. Another study used the Bruker MALDI Biotyper to identify isolates belonging to the African clade III [46]. Isolates form the South American clade IV were reported to be correctly identified with the Bruker MALDI Biotyper [3,47]. However, as it was not the objective to evaluate the performance of the MALDI-TOF MS during the latter three studies, no reference methods for the identification were used, and it is not known how many isolates were misidentified. Using the bioMérieux Vitek MS IVD library version 3.2, 59/61 (96.7%) of Korean isolates could be correctly identified, with a confidence value of ≥75 (East Asian clade II). In addition, it has been shown that 10 CDC reference strains belonging to four clades could be correctly identified by the bioMérieux Vitek MS and Bruker MALDI Biotyper systems [45]. Information about the performance of the identification methods of the possible 5th clade of *C. auris* is not yet available [43]. As only fragmented information and reports about the use of identification methods without comparison to the gold standard methods is available, a comprehensive analysis of a significant number of isolates from all *C. auris* clades, that evaluates the performance of the two MALDI-TOF MS systems and several qPCR methods, is warranted. 

To aid the rapid identification of *C. auris*, many molecular methods have been developed. They are potentially of use in an outbreak situation, as most are sensitive enough to be used on direct material and do not require a culture, in contrast to MALDI-TOF MS. The published papers that have evaluated the molecular methods to detect and identify *C. auris* have been reviewed elsewhere [25,48]. 

To our knowledge, to date only two cases of *C. auris* colonization have been identified in The Netherlands [49]. As these cases were imported from endemic regions, it seems critical that identification methods are used that can accurately identify all *C. auris* clades, in order not to miss cases. The screening of patients for *C. auris* was recently recommended by an expert panel, indicating that laboratory protocols should include the speciation of colonizing *Candida* isolates. Identified risk groups include patients admitted previously to an ICU in an endemic country, and transfers from hospitals that are known to have *C. auris*. When *C. auris* is detected, an infection prevention control that is measured specifically for *C. auris* should be implemented in a timely manner, to prevent (further) spread [32].

## 5. Conclusions

Several laboratories in The Netherlands were not able to correctly identify *C. auris* isolates. Only 2/14 laboratories that used methods which could result in the misidentification of *C. auris* mentioned that they would perform additional testing, or send isolates to reference laboratories, to exclude *C. auris* when needed. However, as 45/47 of participating laboratories have a MALDI-TOF MS system available for the identification of yeast species, and the most used MALDI-TOF MS libraries in The Netherlands currently contain several *C. auris* spectra, we expect that the ability to identify *C. auris* in The Netherlands has increased significantly. 

## Figures and Tables

**Table 1:** Identification of *C. auris*, *C. haemulonii* complex, and *C. lusitatiae* by two MALDI-TOF MS systems.

Isolate	Bruker MALDI Biotyper	bioMérieux. Vitek MS
*C. haemulonii* complex	32/33	10/12
*C. auris*	32/33	3/12 ^1^
*C. lusitaniae*	33/33	12/12
*C. auris*	32/33	3/12 ^1^
*C. haemulonii* complex	33/33	12/12
*C. auris*	32/33	3/12 ^1^

^1^ Identified as *C. auris* with additional methods.

**Table 2:** Identification of *C. auris, C. haemulonii* complex, and *C. lusitatiae* by university and community hospitals.

Isolate	University Hospital	Community Hospitals
*C haemulonii* complex	6/7	38/40
*C. auris*	6/7	30/40
*C. lusitaniae*	7/7	40/40
*C. auris*	6/7	30/40
*C haemulonii* complex	7/7	40/40
*C. auris*	6/7	30/40
